# Aberrant gray matter volume and functional connectivity in Parkinson’s disease with minor hallucination

**DOI:** 10.3389/fnagi.2022.923560

**Published:** 2022-09-14

**Authors:** Min Zhong, Chenglin Li, Hongquan Lu, Donghui Xue, Yaxi Wang, Yinyin Jiang, Sha Zhu, Ruxin Gu, Xu Jiang, Bo Shen, Jun Zhu, Wenbin Zhang, Yang Pan, Jun Yan, Li Zhang

**Affiliations:** ^1^Department of Geriatric Neurology, Affiliated Brain Hospital of Nanjing Medical University, Nanjing, China; ^2^Department of Radiology, Affiliated Brain Hospital of Nanjing Medical University, Nanjing, China; ^3^Department of Neurosurgery, Affiliated Brain Hospital of Nanjing Medical University, Nanjing, China; ^4^Institute of Neuropsychiatric Diseases, Affiliated Brain Hospital of Nanjing Medical University, Nanjing, China

**Keywords:** Parkinson’s disease, minor hallucination, gray matter volume, functional connectivity, default mode network

## Abstract

**Background:**

Minor hallucination (MH) is the most common psychotic symptom in Parkinson’s disease (PD); it can develop into well-structured visual hallucination (VH), suggesting that MH may be a staccato form of well-structured VH. However, it remains unclear whether the pathogenesis is the same. Therefore, the aim of this study was to investigate the altered gray matter volume (GMV) and functional connectivity (FC) of MH in PD to further understand the complex mechanisms.

**Materials and methods:**

We included 67 PD patients who attended the outpatient clinic of Nanjing Medical University Affiliated Brain Hospital and recruited 31 healthy controls (HC). Demographic data and clinical characteristics of all subjects were recorded, and cranial structural magnetic resonance imaging (MRI) and resting-state functional MRI data were acquired. Patients were classified into the PD with MH (PD-MH) group and PD without hallucinations or delusions (PD-NH) group. Voxel-based morphometry was used to analyze the differences in GMV in the structural pattern. Seed-based FC was used to analyze the functional pattern. Gaussian random field correction was used, with voxel level *P* < 0.001 and cluster level *P* < 0.05 representing statistically significant differences. Finally, the correlation between FC values and scores on the clinical characteristics assessment scale was analyzed.

**Results:**

In the GMV analysis, compared to the PD-NH group, the PD-MH group had reduced GMV in the medial superior frontal gyrus (SFGmed). In the FC analysis, the FC between the SFGmed and the left middle occipital gyrus and right calcarine sulcus decreased in the PD-MH group compared with the PD-NH group, while the FC between SFGmed and the left middle temporal gyrus increased. Correlation analysis revealed that the FC values of the SFGmed and right calcarine sulcus were correlated with the assessment scores for anxiety and sleep symptoms. The FC values of the SFGmed and left middle occipital gyrus were correlated with assessment scores for rapid eye movement disorder.

**Conclusion:**

The aberrant structure and function of the default mode network and visual processing areas seems to facilitate the generation of MH in PD, as the alteration was previously found in well-structured VH, suggesting that the two hallucinations have similar pathophysiological mechanisms.

## Introduction

Parkinson’s disease (PD) is the second most common neurodegenerative disease after Alzheimer’s disease (Poewe et al., 2017). Throughout the course of PD, more than 75% of patients develop psychosis associated with Parkinson’s disease (PDPsy) (Barrett et al., 2017). Minor hallucination (MH) expands the spectrum of PDPsy, which includes three phenomena: presence hallucination, passage hallucination, and visual illusion (Lenka et al., 2019). A presence hallucination is when a patient feels the presence of someone nearby while focusing on something. A passage hallucination is when the patient glimpses a faint shadow passing by, often identified as a person or animal. The visual illusion includes object misidentification illusion (seeing something as another object of similar shape), pareidolia (seeing a human face in a complex pattern), and kineptosia (seeing a stationary object as a moving object).

As the most common and earliest psychiatric symptom in PD, appearing even before motor symptoms (Pagonabarraga et al., 2016), MH deserves extensive attention. MH is an early predictor of severe complex well-formed visual hallucinations (VH) and dementia (Lenka et al., 2019), and promises to be a clinical marker in the prodromal phase of neurodegenerative diseases (Sumi et al., 2022). Although it is currently recognized that MH is closely related to well-structured VH (Bejr-Kasem et al., 2019), the understanding of MH is incomplete.

Specifically, the pathophysiological mechanisms of hallucination are not well understood. Currently, many hypotheses have emerged to explain the mechanism of hallucinations, including the following: (1) Activation, input, and modulation (Diederich et al., 2004): This model suggests that reduced visual acuity, defective visual information processing, intrusion of dreams during rapid eye movement sleep, and dopaminergic stimulation of the limbic system of the midbrain are all responsible for the generation of VH; in essence, it is a dysregulation between the regulatory control of external and internal visual information production. (2) Perception and attention deficit (Collerton et al., 2005): This model believes that the generation of VH originates from visual information processing defects in the “bottom-up” and “top-down” directions, with the “bottom-up” being the perception of visual information and the “top-down” being the cognitive and attentional processes. (3) Dysfunction of attentional control networks (Shine et al., 2011): This model argues that VH in PD reflects the inability to activate the dorsal attention network (DAN) normally in the presence of ambiguous perception, resulting in an overreliance on the default mode network (DMN) and the ventral attention network (VAN), which leads to internal information insertion and excessive attention to salient features, eventually producing hallucinations. These hypotheses have one thing in common; that is, they all believe that patients with VH have dysfunctional sensation and attention.

Previous studies on the mechanisms of VH have mostly failed to distinguish between well-structured VH and MH, and thus, the findings may vary. Although longitudinal studies have found a temporal association between the two, it is unclear whether they share the same mechanism, and whether those proposed models can be used to explain MH. Some morphometric studies have found similar structural and functional characteristics between MH and well-structured VH (Pagonabarraga et al., 2014; Bejr-Kasem et al., 2019), while overlapping but graded patterns of structural and functional abnormalities have also been found (Marques et al., 2022).

Using cranial magnetic resonance imaging (MRI) data to investigate structural and functional alterations is beneficial for both diagnostic biomarker research and therapeutic evaluation (Wang et al., 2022) and has greatly advanced our understanding of the pathophysiological mechanisms of PD-MH. In addition to structural abnormalities in certain brain areas, aberrant functional connections and network interactions also contribute to the development of hallucinations. Only a few articles have been published on imaging studies of MH (Llebaria et al., 2010; Nishio et al., 2018; Bejr-Kasem et al., 2019, 2021; Sawczak et al., 2019; Murakami et al., 2021), mostly single-modal studies. However, the human brain is a structurally and functionally complex network, and thus, multimodal MRI studies are more advantageous.

In this study, we proposed to analyze the structural and functional alterations in patients with PD with MH with the aim of further exploring the pathophysiological mechanisms of MH.

## Materials and methods

### Participants

From October 2020 to July 2021, we recruited 67 patients with PD from Nanjing Medical University Affiliated Brain Hospital who were diagnosed by two specialists in movement disorder disease. The inclusion criteria were as follows: (1) patients meeting the diagnostic criteria of the United Kingdom PD Brain Bank (Hughes et al., 1992); (2) 45–75 years of age; (3) normal visual acuity or corrected visual acuity; and (4) right-handedness to ensure that the dominant hemisphere is on the left side and to facilitate unified brain function analysis. Patients were initially screened for psychotic symptoms according to the second question “Hallucinations and Psychosis” in Part I of the Movement Disorders Society-Uniform Parkinson’s Disease Rating Scale (Goetz et al., 2008): option 0 was no psychotic symptoms, option 1 was MH, option 2 was well-structured VH with insight, option 3 was well-structured VH without insight, and option 4 was delusions. Patients who selected option 1 and whose MH had appeared stable in the last month were included in the PD with MH (PD-MH) group, whereas those who selected option 0 were included in the PD without hallucinations or delusions (PD-NH) group. The exclusion criteria were as follows: (1) abnormal signals such as intracranial occupancy on cranial MRI and a clear history of cranial trauma and surgery; (2) dementia, based on a Montreal Cognitive Assessment (MoCA) score ≤ 20 (Gill et al., 2008; Dalrymple-Alford et al., 2010); (3) primary psychiatric disorders including schizophrenia and bipolar disorder according to DSM-5 criteria, or current or previous use of antipsychotic medication; and (4) other serious medical diseases. Four patients were excluded for malignant tumors, cerebral atrophy, obstructive hydrocephalus, and dementia. In addition, 31 age- and sex-matched healthy controls (HC) were enrolled in the same period.

This study was approved by the Ethics Committee of the Nanjing Medical University Affiliated Brain Hospital (ethics number: 2020-KY043-01). In accordance with the Declaration of Helsinki, all patients signed an informed consent form before enrollment.

### Clinical evaluation

A structured questionnaire was used to collect general information such as name, gender, age, and education of all subjects and to record in detail the disease duration and the PD medication taken. The levodopa equivalent daily dose (LEDD) was calculated based on the use of anti-PD medicine (Tomlinson et al., 2010). All subjects were scored on the MoCA, rapid eye movement sleep behavior disorder screening questionnaire (RBDSQ) (Stiasny-Kolster et al., 2007), Hamilton Rating Scale for Anxiety (HAMA), and Hamilton Rating Scale for Depression (HAMD), which were used to assess cognition, rapid eye sleep behavior (RBD), anxiety, and depression, respectively. Evaluation of the Unified PD Rating Scale III (UPDRS III), Hoehn–Yahr stage (H–Y stage), and Parkinson’s disease sleep scale (PDSS) (Chaudhuri et al., 2002) for all PD patients was performed to assess motor symptoms and sleep conditions. As mentioned, a questionnaire (Zhong et al., 2021) was used to record the characteristics of MH in the PD-MH group in more detail.

### Magnetic resonance imaging data acquisition

A 3.0T MRI imaging system (Siemens, Germany) with a 64-channel phased-array head coil was used to scan all subjects at Nanjing Medical University Affiliated Brain Hospital. All patients were in the off period, which was specifically defined as the fasting state in the morning, with the anti-PD medicines stopped for at least 24 h, and the sustained-release tablet stopped for at least 72 h. Subjects were asked to lie flat in the MRI scanner with their eyes closed and their whole body relaxed and to stay quiet and awake. For every subject, sponge pads were used to limit head movement, and ear plugs were used to reduce the effects of noise.

The T1-weighted three-dimensional magnetization prepared rapid acquisition gradient echo sequences were used for structural MRI scanning with the following parameters: repetition time (TR) of 1900 ms, echo time (TE) of 2.5 ms, slice thickness of 1 mm, slice number of 176, slice gap of 0 mm, matrix size of 256 × 256, field of view (FOV) of 250 mm × 250 mm, flip angle (FA) of 9°, time point of 192, and the total scan time was 4 min 18 s. The resting-state functional MRI (rs-fMRI) imaging was acquired with the echo planar imaging sequence, with the following parameters: interleaved scanning, TR of 2000 ms, TE of 25 ms, slice thickness of 4 mm, slice number of 33, slice gap of 0 mm, matrix size of 64 × 64, FOV of 240 mm × 240 mm, FA of 9°, time point of 240, and the total scan time was 8 min 6 s.

### Magnetic resonance imaging data preprocessing

#### T1 data

The DICOM format of the raw data was converted to NIFTI format using dcm2nii^1^ software before preprocessing, and the images after reorientation were selected. The T1 images were preprocessed in the MATLAB R2013b environment using Statistical Parametric Mapping software 8 (SPM8^2^) with the following steps: (1) The image quality was examined; (2) VBM8 was used to implement tissue segmentation, dividing the image into gray matter, white matter, and cerebrospinal fluid; (3) using the Diffeomorphic Anatomical Registration Through Exponentiated Lie (DARTEL) method, the images were spatially normalized by matching them to a standard brain template produced by the Montreal Neuroscience Institute, Canada, namely, the MNI standard spatial template, with a voxel size of 1.5 mm × 1.5 mm × 1.5 mm; (4) a normalized gray matter volume (GMV) map was extracted; (5) the image quality was examined again; (6) smoothing was performed using a Gaussian filter of 6mm and 8 mm full width at half maximum (FWHM).

#### Resting-state functional magnetic resonance imaging data

The DICOM format of the raw data was converted to NIFTI format using dcm2nii before preprocessing. The SPM-based data preprocessing software RESTplus V1.2^3^ was used in the MATLAB R2013b environment. The preprocessing steps included the following: (1) removal of the first 10 time points with unstable signals; (2) slice timing: removal of time differences among scans at different levels; (3) realigning: estimation of the head motion and removal of 5 patients for head motion translations > 3 mm or rotations > 3°; (4) spatial normalization: use of a two-step alignment method with a voxel size of 3 mm × 3 mm × 3 mm; (5) smoothing: use of a Gaussian filter of 6 mm FWHM to reduce the effect of affine transformation; (6) detrending: elimination of linear trends; (7) filtering: performed with a retention bandwidth of 0.01–0.08 Hz; (8) nuisance covariates regression: regression on white matter signal, cerebrospinal fluid signal, and 6 head motion parameters.

### Magnetic resonance imaging data analysis

Statistical analysis was performed on the smoothed GMV of the PD-MH, PD-NH, and HC groups, and the brain region of the GMV difference between the PD-MH and PD-NH groups was defined as a region of interest (ROI). The mean time series of the ROI were extracted, and a voxel-wise FC analysis was performed by calculating the temporal correlation between the mean time series of the ROI and the time series of each voxel within the whole brain. The Fisher z transformation was used to normalize the correlation values to obtain normalized *z*-score maps. The *z*-score maps of the three groups were statistically analyzed to obtain the difference in brain regions between the PD-MH and PD-NH groups, and the FC values between these regions and the ROI were extracted and correlated with the scale scores. In addition, we repeated the entire MRI data analysis, changing the isotropic Gaussian kernel from 8 to 6 mm in the smoothed processed portion of the gray matter images. To minimize bias, grouping information was blind to data analysts.

### Statistical analysis

Statistical analysis was performed using IBM SPSS 23.0 software. Measurement data are expressed as the mean ± standard deviation, and the Kolmogorov–Smirnov test was used to analyze the normality of measurement data. For data that conformed to a normal distribution, a two-sample *t*-test was used for pairwise comparisons, and one-way ANOVA was used for three-group comparisons. For non-normally distributed data, the Mann–Whitney U-test was used for pairwise comparisons, and the Kruskal–Wallis H-test was used for three-group comparisons. Categorical data were compared using the Chi-square test. *P* < 0.05 was used to indicate statistically significant differences.

Statistical analysis of MRI data was performed using DPABI V5.0_201001 software^4^ (Yan et al., 2016). In the voxel-based morphometry (VBM) and FC analyses, all three groups were compared using analysis of covariance (ANCOVA), *post hoc* tests using two-sample *t*-tests, and Gaussian random field (GRF) correction for multiple comparisons correction, and voxel level *P* < 0.001 and cluster level *P* < 0.05 were considered statistically significant differences. The covariates in the VBM analysis were age, gender, education, PDSS score, HAMA score, HAMD score, and RBDSQ score, whereas the covariates in the FC analysis were age, gender, education, PDSS score, HAMA score, HAMD score, RBDSQ score, and GMV.

Spearman’s correlation analysis between the FC values and the PDSS and RBDSQ scores were evaluated in the PD-MH group using IBM SPSS 23.0 with a significance threshold of *P* < 0.05, and the results were plotted using GraphPad Prism 8.0.1.

## Results

### Comparison of demographic and clinical data

A total of 98 subjects were included in this study, among which four patients were excluded due to other diseases and five patients were excluded due to excessive head movement. A total of 89 subjects were finally enrolled, including 23 in the PD-MH group, 35 in the PD-NH group, and 31 in the HC group. As shown in Table 1, the mean duration of MH in the MH group was 2.35 years. There were no significant differences between the three groups of subjects in terms of age, gender, education, or MoCA scale scores, while the differences in RBDSQ, HAMA, and HAMD scale scores were significant. The differences between the PD-MH and PD-NH groups were not significant in terms of disease duration, LEDD, UPDRS III scores, and H-Y staging, while the differences in PDSS scores were significant.

**TABLE 1:** Comparison of demographic information and scale data.

	PD-MH (*n* = 23)	PD-NH (*n* = 35)	HC (*n* = 31)	χ^2^/t/Z/F	*P*
Age, year, mean ± SD	60.57 ± 8.88	61.06 ± 8.35	60.35 ± 7.68	0.063	0.939^a^
Gender, male/female	6/17	13/22	12/19	1.063	0.588^b^
Education, year, mean ± SD	10.35 ± 3.77	8.63 ± 4.26	8.55 ± 2.90	4.068	0.131^c^
MoCA, mean ± SD	26.00 ± 4.70	25.46 ± 4.18	25.84 ± 3.38	0.91	0.634^c^
HAMA, mean ± SD	9.04 ± 4.54	7.54 ± 5.08	2.71 ± 3.21	27.768	**< 0.001** ^c^
HAMD, mean ± SD	9.70 ± 4.49	8.77 ± 4.96	2.74 ± 2.97	36.895	**< 0.001** ^c^
RBDSQ, mean ± SD	4.57 ± 2.81	3.03 ± 2.55	1.81 ± 1.96	13.458	**0.001** ^c^
RBD, *n* (%)	18 (78.26)	12 (34.29)	0 (0.00)	36.199	**< 0.001** ^b^
H–Y stage, mean ± SD	2.59 ± 0.70	2.31 ± 0.58	NA	340.5	0.299^d^
UPDRS III, mean ± SD	32.78 ± 13.28	30.97 ± 13.74	NA	366.5	0.567^d^
PDSS, mean ± SD	97.65 ± 19.22	114.40 ± 20.67	NA	201.5	**0.001** ^d^
LEDD, mg, mean ± SD	655.46 ± 306.44	459.35 ± 502.62	NA	1.675	0.1^e^00
Disease duration, year, mean ± SD	7.08 ± 5.12	5.06 ± 4.11	NA	1.66	0.102^e^
Duration of MH, year, mean ± SD	2.35 ± 1.39	NA	NA	NA	NA

^a^One-way ANOVA, ^b^Chi-square test, ^c^Kruskal–Wallis H-test, ^d^Mann–Whitney U-test, ^e^two-sample t-test. p < 0.05 was statistically different and was shown in bold. SD, standard deviation; PD, Parkinson’s disease; MH, minor hallucination; NH, no hallucinations (and delusions); HC, healthy control; MoCA, Montreal Cognitive Assessment; HAMA, Hamilton Rating Scale for Anxiety; HAMD, Hamilton Rating Scale for Depression; RBDSQ, rapid eye movement sleep behavior disorder screening questionnaire; RBD, rapid eye movement sleep behavior disorder; H–Y stage, Hoehn–Yahr stage; UPDRS III, Unified Parkinson’s Disease Rating Scale Part III; PDSS, Parkinson’s disease sleep scale; LEDD, levodopa equivalent daily dose; NA, not applicable.

### Comparison of gray matter volume

**FIGURE 1:**
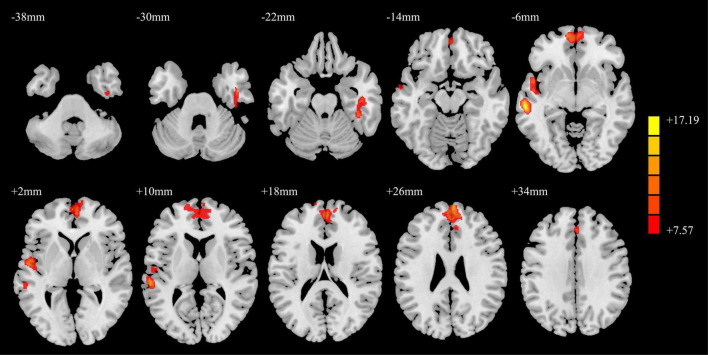
ANCOVA results of gray matter volumes in the PD-MH, PD-NH, and HC groups. Red and yellow indicated altered gray matter volume. Using GRF correction, voxel level *P* < 0.001 and cluster level *P* < 0.05 were considered statistically significant difference. ANCOVA: analysis of covariance; PD, Parkinson’s disease; MH, minor hallucination; NH, no hallucinations (and delusions); HC, healthy control; GRF, Gaussian random field.

The results of the GMV analysis among the three groups are shown in Figure 1. The GMV of the right fusiform gyrus, right inferior temporal gyrus, left middle temporal gyrus, bilateral medial superior frontal gyrus (SFGmed), and left superior temporal gyrus changed.

**FIGURE 2:**
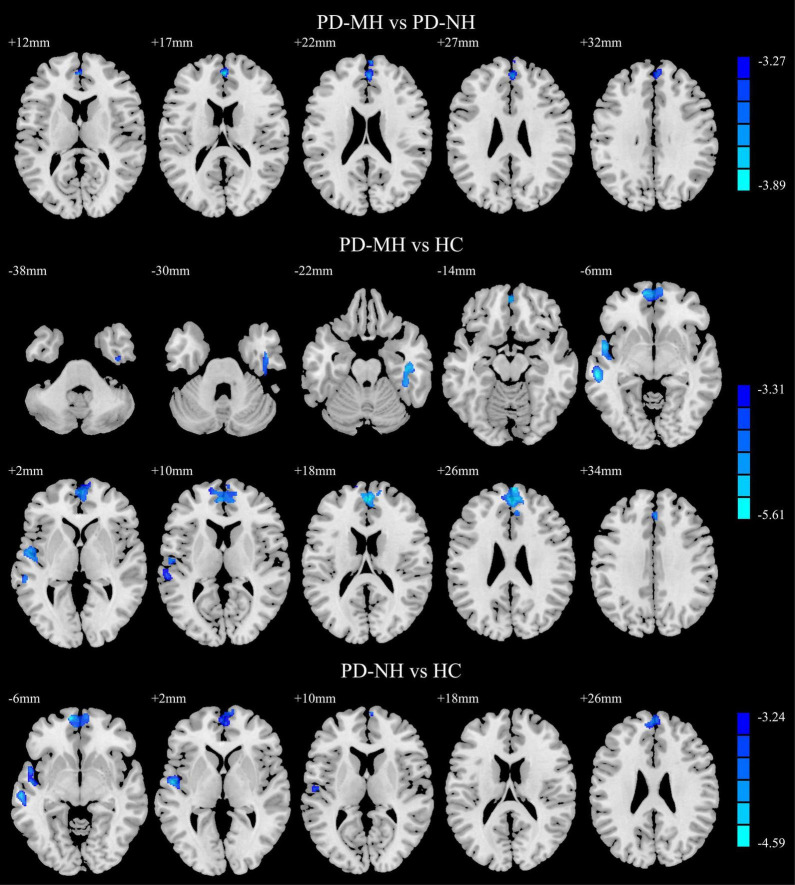
Significant differences of gray matter volume between the groups. Blue and green indicated the reduced gray matter volume. Using GRF correction, voxel level *P* < 0.001 and cluster level *P* < 0.05 were considered statistically significant difference. PD, Parkinson’s disease; MH, minor hallucination; NH, no hallucinations (and delusions); HC, healthy control; GRF, Gaussian random field.

The results of the *post hoc* analysis are shown in Figure 2. Compared with the PD-NH group, the PD-MH group had decreased GMV in the bilateral SFGmed. Compared with the HC group, the PD-MH group displayed lower GMV in the right fusiform gyrus, right inferior temporal gyrus, left middle temporal gyrus, left superior temporal gyrus, and bilateral SFGmed; the PD-MH group displayed lower GMV in the bilateral orbital SFGmed, left middle temporal gyrus, left superior temporal gyrus, and left SFGmed (see Table 2 for details). The atrophy pattern found in patients with MH remained similar when FWHM was changed (see Supplementary Figure 1 and Supplementary Table 1).

**TABLE 2:** Comparison of gray matter volume in PD-MH group, PD-NH group, and HC group.

Brain region	Peak MNI coordinate			Cluster size	F/t
	
	*x*	*y*	*z*		
**ANCOVA**					
Fusiform_R/Temporal_Inf_R	45	–7.5	–33	758	11.3817
Temporal_Mid_L	–60	–27	–6	933	17.1861
Frontal_Sup_Medial_B	3	57	22.5	3290	15.1296
Temporal_Sup_L	–55.5	–7.5	4.5	819	13.0167
**PD-MH vs. PD-NH**					
Frontal_Sup_Medial_B	0	49.5	18	555	–3.8918
**PD-MH vs. HC**					
Fusiform_R/Temporal_Inf_R	42	–19.5	–21	753	–5.0515
Frontal_Sup_Medial_B	3	63	24	3179	–5.6106
Temporal_Mid_L	–60	–25.5	–6	723	–5.3870
Temporal_Sup_L	–54	3	–3	810	–5.4034
**PD-NH vs. HC**					
Frontal_Med_Orb_B	–9	60	–7.5	935	–4.5907
Temporal_Mid_L	–61.5	–21	–7.5	273	–4.4345
Temporal_Sup_L	–54	–7.5	3	736	–4.2956
Frontal_Sup_Medial_L	1.5	57	22.5	326	–4.0598

Three groups were compared using analysis of covariance, and two groups were compared using a two-sample t-test. Differences were statistically significant using GRF correction, voxel level P < 0.001, and cluster level P < 0.05. MNI, Montreal Neuroscience Institute; PD, Parkinson’s disease; MH, minor hallucination; NH, no hallucinations (and delusions); HC, healthy control; ANCOVA, analysis of covariance; GRF, Gaussian random field.

### Comparison of functional connectivity

**FIGURE 3:**
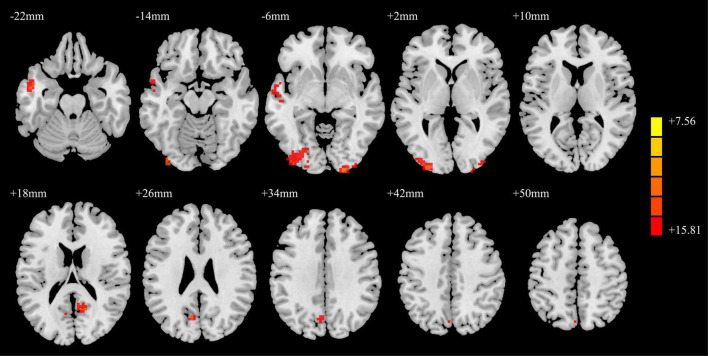
ANCOVA results of functional connectivity in the PD-MH, PD-NH, and HC groups. Red and yellow indicated altered functional connectivity. Using GRF correction, voxel level *P* < 0.001 and cluster level *P* < 0.05 were considered statistically significant difference. ANCOVA, analysis of covariance; PD, Parkinson’s disease; MH, minor hallucination; NH, no hallucinations (and delusions); HC, healthy control; GRF, Gaussian random field.

The SFGmed was used as a seed point for voxel-wise FC to the whole brain. The ANCOVA result is shown in Figure 3. The differences in SFGmed FC for brain regions among the PD-MH, PD-NH, and HC groups were observed in the left middle temporal gyrus, right inferior occipital/calcarine/middle occipital gyrus, left middle occipital/inferior occipital gyrus, right precuneus, and left cuneus.

**FIGURE 4:**
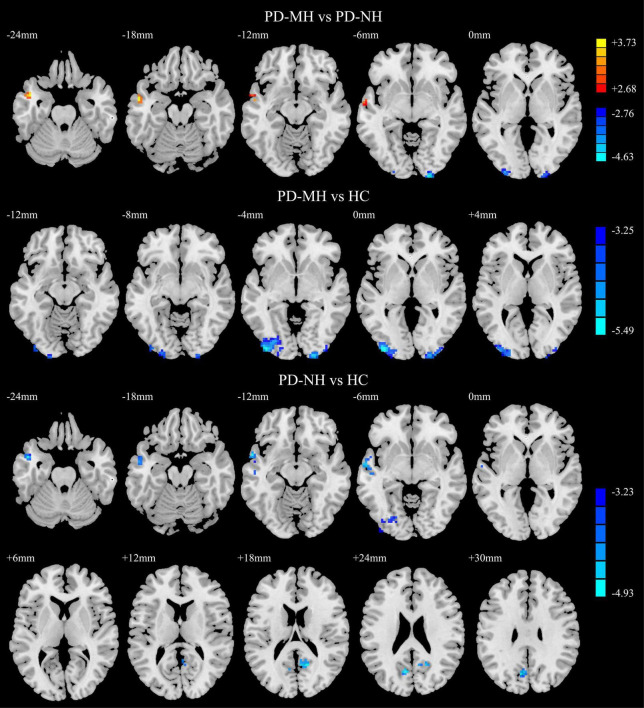
Significant differences of functional connectivity between the groups. Red and yellow indicated increased functional connectivity. Blue and green indicated reduced functional connectivity. Using GRF correction, voxel level *P* < 0.001 and cluster level *P* < 0.05 were considered statistically significant difference. PD, Parkinson’s disease; MH, minor hallucination; NH, no hallucinations (and delusions); HC, healthy control; GRF, Gaussian random field.

As shown in Figure 4, compared with the PD-NH group, the PD-MH group had enhanced FC between the SFGmed and left middle temporal gyrus and decreased FC between the SFGmed and brain regions such as the right calcarine sulcus and left middle occipital gyrus. Compared with the HC group, the PD-MH group showed decreased FC between the SFGmed and the left middle occipital/inferior occipital gyrus and right inferior occipital/calcarine/middle occipital gyrus. The PD-NH group showed decreased FC between the SFGmed and the left middle temporal gyrus, left inferior occipital gyrus, right precuneus, and left cuneus (see Table 3 for details). The connectivity pattern found in patients with MH remained similar when FWHM was changed (see Supplementary Figure 2 and Supplementary Table 2).

**TABLE 3:** Comparison of functional connectivity in PD-MH group, PD-NH group, and HC group.

Brain region	Peak MNI coordinate			Cluster size	F/t
	
	*x*	*y*	*z*		
**ANCOVA**					
Temporal_Mid_L	–48	3	24	88	11.9443
Occipital_Inf_R/Calcarine_R/Occipital_Mid_R	21	–99	–3	58	15.3276
Occipital_Mid_L/Occipital_Inf_L	–39	–90	–15	162	13.8536
Precuneus_R	12	–57	18	42	11.6281
Cuneus_L	–6	–69	30	44	11.0454
**PD-MH vs. PD-NH**					
Temporal_Mid_L	–45	3	–24	47	3.7301
Calcarine_R	21	–102	–6	30	–4.6331
Occipital_Mid_L	–30	–96	0	28	–3.9074
**PD-MH vs. HC**					
Occipital_Mid_L/Occipital_Inf_L	–39	–90	0	153	–5.4881
Occipital_Inf_R/Calcarine_R/Occipital_Mid_R	24	–99	–3	56	–5.0148
**PD-NH vs. HC**					
Temporal_Mid_L	–57	6	–12	87	–4.8034
Occipital_Inf_L	–27	–81	–3	35	–3.8773
Precuneus_R	12	–57	21	42	–4.8661
Cuneus_L	–9	–66	24	44	–4.9340

Three groups were compared using analysis of covariance, and two groups were compared using a two-sample t-test. Differences were statistically significant using GRF correction, voxel level P < 0.001, and cluster level P < 0.05. MNI, Montreal Neuroscience Institute; PD, Parkinson’s disease; MH, minor hallucination; NH, no hallucinations (and delusions); HC, healthy control; ANCOVA, analysis of covariance; GRF, Gaussian random field.

### Correlation analysis

**FIGURE 5:**
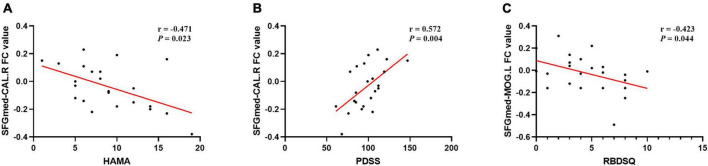
Correlation analysis of FC and clinical scale data in MH patients. A significant correlation between **(A)** SFGmed-CAL.R FC value and HAMA score (*rs* = –0.471, *P* = 0.023); **(B)** SFGmed-CAL.R FC value and PDSS score (*rs* = 0.572, *P* = 0.004); **(C)** SFGmed-MOG.L FC value and RBDSQ score (*rs* = –0.423, *P* = 0.044); PD, Parkinson’s disease; MH, minor hallucination; FC, functional connectivity; SFGmed, medial superior frontal gyrus; CAL.R, right calcarine sulcus; MOG.L, left middle occipital gyrus; HAMA, Hamilton Rating Scale for Anxiety; PDSS, Parkinson’s disease sleep scale; RBDSQ, rapid eye movement sleep behavior disorder screening questionnaire.

The results of correlation analysis are shown in Table 4 and Figure 5. In the PD-MH group, FC strength between the SFGmed and the right calcarine sulcus showed a negative relationship with HAMA score and a positive relationship with PDSS score. FC strength between the SFGmed and the left middle occipital gyrus showed a negative relationship with RBDSQ score. No significant correlation was seen between the connection of the SFGmed and the left middle temporal gyrus with assessment scale scores. When FWHM was changed, the correlation between the FC strength and RBDSQ score was not statistically significant (see Supplementary Table 3).

**TABLE 4:** Correlation analysis between FC values and scale scores in PD-MH group.

	SFGmed-CAL.R FC value		SFGmed-MOG.L FC value		SFGmed-MTG.L FC value	
			
	*r*	*P*	*r*	*P*	*r*	*P*
MoCA	–0.407	0.054	–0.314	0.145	0.062	0.778
RBDSQ	–0.257	0.237	–0.423	**0.044**	–0.388	0.067
HAMA	–0.471	**0.023**	–0.390	0.066	–0.409	0.053
HAMD	–0.371	0.081	–0.202	0.355	–0.152	0.488
H–Y stage	0.043	0.846	–0.133	0.544	–0.038	0.863
UPDRS III	–0.091	0.680	–0.216	0.322	–0.021	0.925
PDSS	0.572	**0.004**	0.366	0.086	0.210	0.337
PDQ39	–0.317	0.140	–0.328	0.126	–0.230	0.290
Duration of MH	0.219	0.314	0.051	0.819	–0.042	0.848

Spearman’s correlation analysis was used. p < 0.05 was statistically different and was shown in bold. PD, Parkinson’s disease; MH, minor hallucination; FC, functional connectivity; SFGmed, medial superior frontal gyrus; CAL.R, right calcarine sulcus; MOG.L, left middle occipital gyrus; MTG.L, left middle temporal gyrus; MoCA, Montreal Cognitive Assessment; RBDSQ, rapid eye movement sleep behavior disorder screening questionnaire; HAMA, Hamilton Rating Scale for Anxiety; HAMD, Hamilton Rating Scale for Depression; H–Y stage, Hoehn–Yahr stage; UPDRS III, Unified Parkinson’s Disease Rating Scale Part III, PDSS: Parkinson’s disease sleep scale.

## Discussion

In this study, we used a combination of VBM and FC to analyze imaging data and found that brain regions in PD-MH patients exhibit focal structural and functional abnormalities. The main findings of this study include the following: patients with MH showed greater loss of GMV in DMN regions (bilateral SFGmed, left middle temporal gyrus, and left superior temporal gyrus) and ventral visual pathways (right cingulate gyrus, right inferior temporal gyrus) than the PD-NH group. The FC between the SFGmed and the bilateral visual network (bilateral middle occipital gyrus, bilateral inferior occipital gyrus, and right calcarine sulcus) was weakened, while the FC to the left middle temporal gyrus was increased. The FC strength between the SFGmed and the left middle occipital gyrus was negatively correlated with the RBDSQ score. The FC strength between the SFGmed and the right calcarine sulcus was positively correlated with the PDSS score, while it was negatively correlated with the HAMA score. In general, the results were stable and reliable, as there were no specific changes due to FWHM alterations. The pattern of GMV reduction in MH patients was consistent with previous studies. Bejr-Kasem et al. (2019) also found atrophy in some brain regions in the DMN and ventral visual pathway when comparing GMV differences between MH and NH patients. Previously, reduced GMV in the prefrontal, cingulate, and inferior temporal gyri were found in patients with well-structured VH (Ibarretxe-Bilbao et al., 2010; Shin et al., 2012; Watanabe et al., 2013; Goldman et al., 2014). These results suggest that cortical involvement is already present in patients with MH, but the extent of GMV loss is less than that of well-structured VH, further confirming that the former is a prodromal stage of the latter and may have the same pathophysiological mechanism.

Medial superior frontal gyrus belongs to the medial prefrontal lobe, which is the center of the anterior DMN. This study further confirmed that the DMN plays an important role in MH generation, and it was complementary to the relative GMV reduction of posterior cingulate-centered post-DMN found in previous studies (Bejr-Kasem et al., 2019). The DMN involves brain regions mainly in the medial prefrontal, posterior cingulate/precuneus, and inferior parietal lobules (Raichle, 2015), whose role is to dynamically integrate external inputs with internal prior knowledge (Yeshurun et al., 2021). It constitutes the complete system required for the cognitive functions of self-reference, self-monitoring, value judgments, and other self-referential mental activities (Menon, 2015), in which the medial prefrontal lobe is differentially linked to self-relevant and social cognitive processes, value-based decision-making, and emotion regulation. Abnormalities in the intrinsic functional connectivity of the DMN have also been observed in Alzheimer’s disease and in almost all major psychiatric disorders (Hiser and Koenigs, 2018). The DMN plays a similarly important role in PD with VH. The model of attention control network dysfunction based on brain network research suggests that hallucinations arise because of DAN dysfunction, resulting in inappropriate activation of the DMN and VAN (Shine et al., 2011, 2014b). PD forms blurred visual information due to the decline in bottom-up visual perception, and because the DAN is dysfunctional, it cannot arouse active attention, so other neural networks are activated to replace DAN executive functions. These activated networks include the DMN (where the patient substitutes past experiences and recollections for what is seen) and the VAN (where the patient focuses attention on the object’s salient stimuli), both of which increase the error rate of visual afferent information. The researchers believe that the attention control network dysfunction model is also applicable to MH (Shine et al., 2011, 2014a). However, our study was unable to draw conclusions about changes in the attentional network. Only one article has reported that the FC between the cingulate gyrus and several regions of the DAN is reduced in PD-MH patients (Bejr-Kasem et al., 2019). Our finding that the decreased GMV of MH patients in bilateral SFGmed lends support to clinical hypotheses about the significance of MH as a risk factor for the development of well-structured VH and psychosis, as well as for a more severe and rapidly advancing form of PD (Bernasconi et al., 2021). Furthermore, since the medial prefrontal cortex is closely related to episodic memory and emotional control, early GMV loss in this region may be associated with the progression of MH to depression and cognitive decline.

The normal visual pathway in the human body runs from the retina of the eye through the lateral geniculate body to the primary visual cortex and is then divided into two visual pathways: one projecting dorsally, involved in spatial and motion perception, and the other projecting ventrally, associated with object recognition and perception (Nishio et al., 2018; Erskine et al., 2019). Involved in the formation of the ventral visual pathway are mainly the inferior temporal gyrus and fusiform gyrus, and we found that MH patients showed reduced GMV in this region, leading to impaired object recognition, which may be responsible for the generation of MH. Previous studies with different modality data, such as MRI, PET, and SPECT, have found reduced activation and metabolism of the ventral visual pathway in patients with VH (Stebbins et al., 2004; Matsui et al., 2006; Meppelink et al., 2009; Park et al., 2013; Firbank et al., 2018).

Abnormalities in the fusiform gyrus play an important role in visual illusion (Kanwisher and Yovel, 2006; Murakami et al., 2021), and Liu et al. (2014) found significant activation of the right fusiform gyrus when patients experienced pareidolia. The involvement of the temporal lobe in the development of VH has been well characterized. In patients with Lewy body disease, there is a strong correlation between Lewy bodies distribution in the temporal lobe and the existence of VH (Harding et al., 2002). Studies have found that Lewy bodies have a relatively greater deposition in the temporal region of the ventral stream and their density increases in a gradient to the anterior temporal lobe; these pathological changes are speculated to contribute to the production of VH (Firbank et al., 2018). The increased connectivity of the SFGmed and the left middle temporal gyrus in the PD-MH group may indicate the compensatory effect of regional atrophy.

Jardri et al. (2013) found that VH was associated with reduced functional connectivity between visual areas and the DMN. Consistent with the findings in VH, we found diminished connectivity between the DMN, represented by the medial prefrontal gyrus, and the visual cortex. The primary visual cortex (calcarine sulcus) and higher visual cortex (occipital gyrus) were found to be involved. The DMN provided a possible point of convergence between bottom-up and top-down modulation theories (Bejr-Kasem et al., 2019). Because of the reduced FC between the visual cortex and the SFGmed, bottom-up visual information afferents are impaired; combined with the cortical release theory (Fernyhough, 2019), there is top-down attentional hyperactivation; therefore, top-down and bottom-up visual information processing impairments can well explain the occurrence of MH (Hepp et al., 2017). In addition, we found that the FC strength of the SFGmed and the left middle occipital gyrus correlated with RBD assessment scores and that the FC strength of the SFGmed and the right calcarine sulcus correlated with PDSS and mood assessment scores. Previous studies have found correlations between MH and RBD, sleep quality, and mood disorder (Zhong et al., 2021); thus, reduced FC between the SFGmed and the visual cortex is important in MH and may be an imaging marker of it. Since MH and the beginning symptoms of RBD such as vivid dreams and sleep disruption were reported to be warning signs of worsen cognitive impairment during the course of PD (Lenka et al., 2016), early identification and intervention of MH are significant for these patients.

The following shortcomings exist in this study: (1) The sample size is relatively small, especially in the PD-MH group, and thus, there may be a statistical error. (2) Because of the need to exclude the effects of medications, the included PD patients rarely included patients with tremor as the predominant phenotype.

## Conclusion

The pathophysiological mechanisms behind hallucinations are the result of the disruption of multiple complex neural circuits rather than individual or discrete lesions. Structural and functional alterations in the DMN and visual processing-related areas are involved in the generation of MH. Top-down and bottom-up visual processing deficits can be used to explain the mechanisms of MH production. Although a simple dichotomy between MH and well-structured VH may help to describe the experience of different hallucinatory phenomena, the boundary between the two types of hallucinations is not always clear. We found that MH and well-structured VH may share the same pathophysiological mechanisms, and the former shows a gradual progression toward the latter in terms of structural and functional changes.

## Data availability statement

The raw data supporting the conclusions of this article will be made available by the authors, without undue reservation.

## Ethics statement

The studies involving human participants were reviewed and approved by the Ethics Committee of the Nanjing Medical University Affiliated Brain Hospital (ethics number: 2020-KY043-01). The patients/participants provided their written informed consent to participate in this study. Written informed consent was obtained from the individual(s) for the publication of any potentially identifiable images or data included in this article.

## Author contributions

LZ, XJ, BS, and MZ conceived and designed the research. LZ, XJ, and JY obtained the funding. MZ, CL, HL, DX, YW, YJ, SZ, RG, YP, WZ, and JZ collected the data. MZ, CL, HL, XJ, and BS conducted the data analysis. MZ drafted the manuscript. All authors contributed to the article and approved the submitted version.

## Footnotes

1
https://people.cas.sc.edu/rorden/mricron/dcm2nii.html

2
http://www.fil.ion.ucl.ac.uk/spm

3
http://www.restfmri.net

4
http://www.rfmri.org/dpabi
