# Supplementary material for: Aberrant gray matter volume and functional connectivity in Parkinson’s disease with minor hallucination
Source: Front Aging Neurosci. 2022 Sep 14;14:923560. doi: 10.3389/fnagi.2022.923560 (PMC9522711; doi:10.3389/fnagi.2022.923560)
Supplement: Supplementary file 1 [file Data_Sheet_1.docx]

Supplementary Information

Includes: Figures S1-S2 and Table S1-S3

Figure S1 Significant differences of gray matter volume across the groups.

Using GRF correction, voxel level P<0.001 and cluster level P<0.05 were considered statistically significant difference. PD: Parkinson's disease, MH: minor hallucination, NH: no hallucinations (and delusions), HC: healthy control, GRF: Gaussian random field.

Table S1 Comparison of gray matter volume in PD-MH group, PD-NH group and HC group

| Brain region | Peak MNI coordinate | | | Cluster size | F/t |
| --- | --- | --- | --- | --- | --- |
|  | x | y | z |  |  |
| ANCOVA | | | | | |
| Fusiform_R/Temporal_Inf_R | 39 | -33 | -19.5 | 516 | 12.719 |
| Temporal_Mid_L | -60 | -27 | -6 | 436 | 17.3367 |
| Frontal_Med_Orb_B | -9 | 60 | -7.5 | 450 | 14.8607 |
| Frontal_Sup_Medial_B | 4.5 | 58.5 | 22.5 | 697 | 16.5573 |
| Temporal_Sup_L | -54 | 7.5 | -4.5 | 563 | 15.8676 |
| PD-MH vs PD-NH | | | | | |
| Frontal_Sup_Medial_B | 0 | 49.5 | 18 | 158 | -4.2415 |
| PD-MH vs HC | | | | | |
| Fusiform_R/Temporal_Inf_R | 42 | -19.5 | -19.5 | 510 | -5.1966 |
| Frontal_Med_Orb_B | -3 | 57 | -3 | 399 | -5.3506 |
| Frontal_Sup_Medial_B | 4.5 | 60 | 22.5 | 697 | -6.0139 |
| Temporal_Mid_L | -60 | -25.5 | -6 | 394 | -5.5884 |
| Temporal_Sup_L | -54 | 6 | -4.5 | 559 | -5.433 |
| PD-NH vs HC | | | | | |
| Frontal_Med_Orb_L | -10.5 | 60 | -7.5 | 249 | -5.071 |
| Frontal_Med_Orb_R | 6 | 66 | 6 | 124 | -4.5858 |
| Temporal_Mid_L | -61.5 | -21 | -7.5 | 203 | -4.4058 |
| Temporal_Sup_L | -52.5 | 7.5 | -4.5 | 459 | -4.7226 |
| Frontal_Sup_Medial_L | 3 | 57 | 22.5 | 174 | -4.3963 |

Note: Three groups were compared using analysis of covariance, and two groups were compared using a two-sample t-test. Differences were statistically significant using GRF correction, voxel level P<0.001 and cluster level P<0.05.

MNI: Montreal Neuroscience Institute; PD: Parkinson's disease; MH: minor hallucination; NH: no hallucinations (and delusions); HC: healthy control; ANCOVA: analysis of covariance; GRF: Gaussian random field.

Figure S2 Significant differences of functional connectivity across the groups.

Using GRF correction, voxel level P<0.001 and cluster level P<0.05 were considered statistically significant difference. PD: Parkinson's disease, MH: minor hallucination, NH: no hallucinations (and delusions), HC: healthy control, GRF: Gaussian random field.

Table S2 Comparison of functional connectivity in PD-MH group, PD-NH group and HC group

| Brain region | Peak MNI coordinate | | | Cluster size | F/t |
| --- | --- | --- | --- | --- | --- |
|  | x | y | z |  |  |
| ANCOVA | | | | | |
| Temporal_Mid_L | -48 | 3 | -21 | 87 | 11.6705 |
| Occipital_Mid_R/Occipital_Inf_R/Calcarine_R | 24 | -102 | -6 | 67 | 15.0968 |
| Occipital_Mid_L/Occipital_Inf_L | -42 | -87 | -3 | 232 | 13.6659 |
| PD-MH vs PD-NH | | | | | |
| Temporal_Mid_L | -51 | -3 | -21 | 44 | 3.776 |
| Calcarine_R | 21 | -102 | -6 | 35 | -4.643 |
| Occipital_Mid_L | -30 | -96 | 0 | 42 | -4.3287 |
| PD-MH vs HC | | | | | |
| Occipital_Mid_L/Occipital_Inf_L | -42 | -90 | -3 | 225 | -5.8788 |
| Occipital_Inf_R/Occipital_Mid_R/Calcarine_R | 24 | -102 | -3 | 63 | -5.0165 |
| PD-NH vs HC | | | | | |
| Temporal_Mid_L | -57 | 6 | -12 | 86 | -4.6534 |
| Occipital_Inf_L | -24 | -78 | -3 | 34 | -4.0421 |

Note: Three groups were compared using analysis of covariance, and two groups were compared using a two-sample t-test. Differences were statistically significant using GRF correction, voxel level P<0.001 and cluster level P<0.05.

MNI: Montreal Neuroscience Institute; PD: Parkinson's disease; MH: minor hallucination; NH: no hallucinations (and delusions); HC: healthy control; ANCOVA: analysis of covariance; GRF: Gaussian random field.

Table S3 Correlation analysis between FC values and scale scores in PD-MH group

|  | SFGmed-CAL.R FC value | | SFGmed-MOG.L FC value | | SFGmed-MTG.L FC value | |
| --- | --- | --- | --- | --- | --- | --- |
|  | r | P | r | P | r | P |
| MoCA | -0.396 | 0.062 | -0.355 | 0.097 | -0.01 | 0.965 |
| RBDSQ | -0.205 | 0.348 | -0.304 | 0.159 | -0.384 | 0.07 |
| HAMA | -0.416 | **0.049** | -0.359 | 0.093 | -0.394 | 0.063 |
| HAMD | -0.385 | 0.07 | -0.194 | 0.375 | -0.126 | 0.566 |
| H-Y stage | 0.058 | 0.794 | -0.179 | 0.413 | -0.216 | 0.322 |
| UPDRS Ⅲ | -0.093 | 0.672 | -0.219 | 0.315 | -0.171 | 0.435 |
| PDSS | 0.648 | **<0.001** | 0.371 | 0.081 | 0.171 | 0.436 |
| PDQ39 | -0.337 | 0.115 | -0.253 | 0.244 | -0.205 | 0.347 |
| Duration of MH | 0.126 | 0.566 | 0.007 | 0.976 | -0.173 | 0.43 |

Note: Spearman's correlation analysis was used. p < 0.05 was statistically different and was shown in bold.

PD: Parkinson's disease; MH: minor hallucination; FC: functional connectivity; SFGmed : medial superior frontal gyrus; CAL.R: right calcarine sulcus; MOG.L: left middle occipital gyrus; MTG.L: left middle temporal gyrus; MoCA: Montreal Cognitive Assessment; RBDSQ: Rapid eye movement sleep Behavior Disorder Screening Questionnaire; HAMA: Hamilton Rating Scale for Anxiety; HAMD: Hamilton Rating Scale for Depression; H-Y stage: Hoehn-Yahr stage; UPDRS Ⅲ : Unified Parkinson's Disease Rating Scale Part III; PDSS: Parkinson's Disease Sleep Scale.
