# Supplementary figures and images for: Aberrant gray matter volume and functional connectivity in Parkinson’s disease with minor hallucination
Source: Front Aging Neurosci. 2022 Sep 14;14:923560. doi: 10.3389/fnagi.2022.923560 (PMC9522711; doi:10.3389/fnagi.2022.923560)

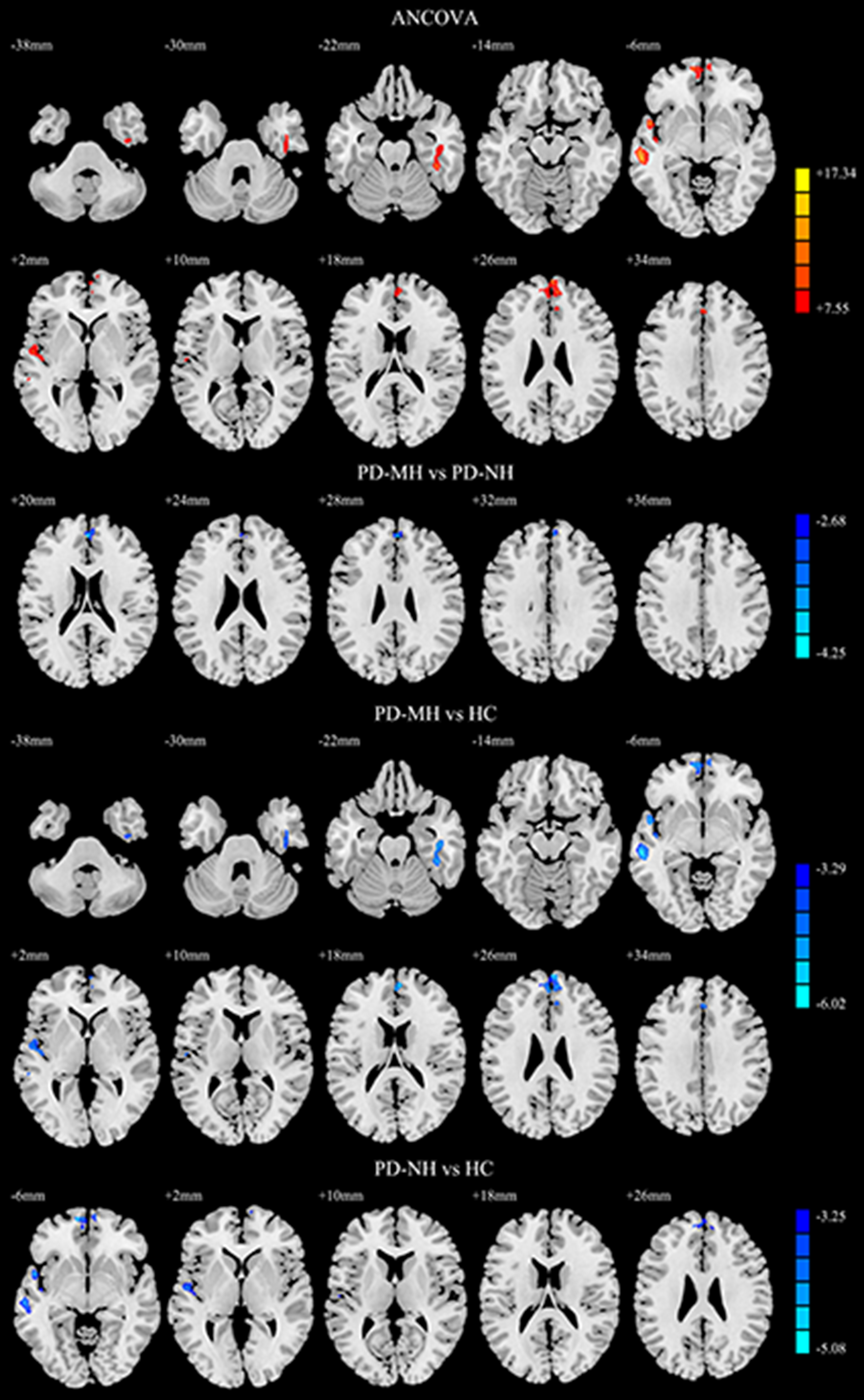

Supplement: Supplementary file 2 [file Image_1.TIF]

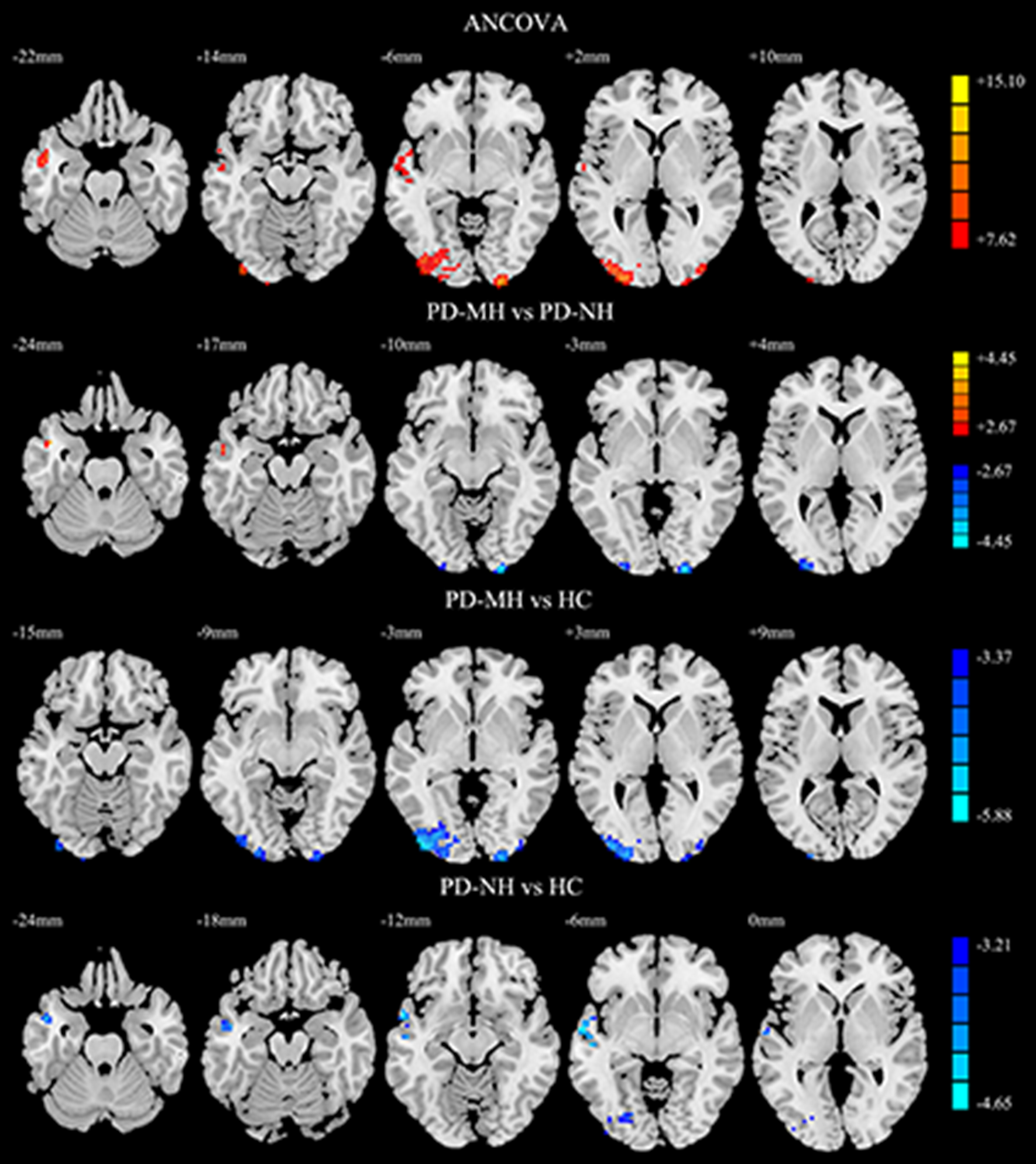

Supplement: Supplementary file 3 [file Image_2.TIF]
